# Draft Genome Sequences of *Pseudoalteromonas tetraodonis* CSB01KR and *Pseudoalteromonas lipolytica* CSB02KR, Isolated from the Gut of the Sea Cucumber *Apostichopus japonicus*

**DOI:** 10.1128/genomeA.00627-17

**Published:** 2017-07-13

**Authors:** Jihoon Jo, Hyunjun Choi, Sung-Gwon Lee, Jooseong Oh, Hyun-Gwan Lee, Chungoo Park

**Affiliations:** a School of the Biological Sciences and Technology, Chonnam National University, Gwangju, Republic of Korea; b Department of Oceanography, Marine Ecological Disturbing and Harmful Organisms Research Center, Chonnam National University, Gwangju, Republic of Korea

## Abstract

We present here the complete genome sequences of two newly isolated *Pseudoalteromonas tetraodonis* and *Pseudoalteromonas lipolytica* strains, isolated from the gut of the sea cucumber *Apostichopus japonicus*, to provide a useful means for facilitating the study of antibacterial, bacteriolytic, agarolytic, and algicidal activities of marine *Pseudoalteromonas* species.

## GENOME ANNOUNCEMENT

The genus *Pseudoalteromonas* belongs to the *Gammaproteobacteria* class, with 38 recognized species so far. *Pseudoalteromonas* spp. are heterotrophic Gram-negative flagellated bacteria commonly found in various ocean habitats, including the deep sea, polar sea, and other extreme marine habitats (1–3). Along with these distinct lifestyles, which highlight their diverse roles in marine ecosystems, *Pseudoalteromonas* species produce biologically active metabolites with antibacterial, bacteriolytic, agarolytic, and algicidal activities (4). *Pseudoalteromonas tetraodonis*, isolated from puffer fish, secretes the neurotoxin tetrodotoxin (5), and antifouling activities by *Pseudoalteromonas lipolytica* affect marine invertebrate larval settlement and metamorphosis (6). Here, we report the complete genome sequences of *Pseudoalteromonas tetraodonis* CSB01KR and *Pseudoalteromonas lipolytica* CSB02KR strains isolated from the gut of the sea cucumber *Apostichopus japonicus* (34.1N, 127.18E; Geomun-do, Yeosu, Republic of Korea).

The isolated cells of each strain were harvested in marine broth at room temperature for 1 day, and genomic DNA was extracted using proteinase K digestion and phenol-chloroform extraction. The genomes of these strains were sequenced using the Illumina HiSeq 4000 platform, employing paired-end reads (151 bp × 2) prepared by Accel-NGS 2S PCR-free library kit (Illumina, San Diego, CA, USA), according to the manufacturer’s protocol. A total of 7,155,540 and 7,760,584 raw reads were generated from *P. tetraodonis* and *P. lipolytica*, respectively, resulting in 294- and 267-fold coverage of the genomes, respectively. The raw reads were preprocessed and trimmed using the Trimmomatic tool (7), in which reads containing adapter sequences, poly-N sequences, and low-quality bases (below a mean Phred score of 15) were removed. All trimmed reads were *de novo* assembled using four different genome assemblers, SOAP*denovo*2 (8), ABySS version 1.9 (9), Velvet version 1.2 (10), and SPAdes version 3.7 (11), under default parameters with a k-mer range of 33 to 99. Last, qualifying contigs from the four assemblies were integrated using CISA version 1.3 (12). The final assembled contigs were evaluated by QUAST version 4.1 (13). The resulting draft genome for *P. tetraodonis* CSB01KR consisted of 49 contigs, covering 3,675,392 bp, with 40.3% G+C content and an *N*_50_ of 196,947 bp. The draft genome for *P. lipolytica* CSB02KR also consisted of 49 contigs, covering 4,387,864 bp, with 41.5% G+C content and an *N*_50_ of 234,255 bp.

Automatic gene annotation was performed using the program Rapid Annotations using Subsystems Technology (RAST; version 2.0) (14). The annotated *P. tetraodonis* CSB01KR genome contains 3,567 genes, including 3,479 protein-coding sequences classified in 456 subsystems, 5 5S rRNAs, 5 short subunit (SSU) rRNAs, 3 long subunit (LSU) rRNAs, and 75 tRNAs. In the *P. lipolytica* CSB02KR genome, 3,984 genes that consist of 3,904 protein-coding sequences classified in 487 subsystems, 2 5S rRNAs, 1 SSU rRNA, 2 LSU rRNAs, and 75 tRNAs were annotated. We believe that the full-genome sequences of the two *Pseudoalteromonas* strains expand the repertoire of genomic information for the genus *Pseudoalteromonas* and will provide important insights into their role within microbial communities.

### Accession number(s).

The raw data of the two strains have been deposited in the NCBI database under the BioProject accession number PRJNA328511. The assembled draft genomes of *P. tetraodonis* and *P. lipolytica* were deposited at GenBank with accession numbers MBMS01000001 to MBMS01000049 and MBSZ01000001 to MBSZ01000049, respectively.
